# Inflammation in atherosclerotic cardiovascular disease: From diagnosis to treatment

**DOI:** 10.1111/eci.70020

**Published:** 2025-03-08

**Authors:** Natalie Arnold, Wolfgang Koenig

**Affiliations:** ^1^ Department of Cardiology University Heart and Vascular Center Hamburg Hamburg Germany; ^2^ German Center for Cardiovascular Research (DZHK) Partner Site Hamburg/Kiel/Luebeck Hamburg Germany; ^3^ Technical University of Munich, School of Medicine and Health, German Heart Centre, TUM University Hospital Munich Germany; ^4^ German Centre for Cardiovascular Research (DZHK) Partner Site Munich Heart Alliance Munich Germany; ^5^ Institute of Epidemiology and Medical Biometry University of Ulm Ulm Germany

**Keywords:** atherosclerotic cardiovascular disease, colchicine, inflammation, hsCRP

## Abstract

**Background:**

Targeting inflammation offers a unique possibility to address residual cardiovascular risk in almost two thirds of all patients with prevalent atherosclerotic cardiovascular disease (ASCVD). However, despite FDA approval and the ESC 2024 Guidelines for the Management of Chronic Coronary Syndrome recommendations to implement low‐dose colchicine (0.5 mg daily) in the secondary prevention of ASCVD patients with residual inflammatory risk, its clinical adoption is still limited. In this regard, a simple screening for elevated high‐sensitive C‐reactive protein (hsCRP) on a routine basis might help to recognize low‐grade inflammation as an important therapeutic target.

**Results:**

Within the present review, we first provide recently published epidemiologic evidence that hsCRP is at least as strong a predictor of future ASCVD events as traditional lipoproteins. Furthermore, we summarize our recent knowledge on currently available strategies to modulate an inflammatory process in ASCVD and critically discuss still open issues regarding the benefit of colchicine therapy in the acute coronary setting or for stroke prevention. In addition, we also briefly touch upon some specific issues of safety related to the long‐term use of colchicine. Finally, we discuss the next diagnostic and therapeutic frontiers in targeting residual inflammatory risk, such as detection of vascular/coronary inflammation by pericoronary fat attenuation or the use of ziltivekimab, a human monoclonal antibody targeting interleukin‐6.

**Conclusion:**

Thus, the integration of interventions aimed at lowering the inflammatory burden in combination with aggressive lipid‐modifying therapy in secondary prevention may hold the potential to further reduce the still substantial burden of ASCVD.

## Graphical abstract

**Inflammation in atherosclerotic cardiovascular disease.** Residual inflammatory risk (RIR), defined by high‐sensitive C‐reactive protein (hsCRP) concentration of ≥2 mg/L, represents a strong predictor of future atherosclerotic cardiovascular disease (ASCVD) events. Although increased hsCRP values ≥2 mg/L might be detected in every three out of five ASCVD patients, RIR is still under‐diagnosed and under‐treated to date, thereby representing a substantial unmet clinical need. Thus, targeting inflammation in combination with aggressive lipid‐modifying therapy in secondary prevention may hold the potential to further reduce the still substantial burden of ASCVD.

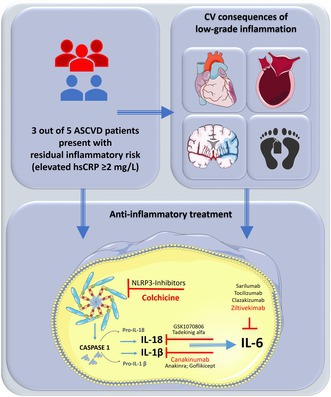

## 1 INTRODUCTION

More than three decades ago Russel Ross postulated the ‘response to injury’ hypothesis, suggesting a tight interplay between lipoproteins and an immune cellular response within the arterial wall as the main trigger of atherosclerotic plaque initiation and characterizing atherosclerosis an ‘inflammatory disease’.^1^ At the same time the first epidemiological evidence has been published on the role of high sensitive C‐reactive protein (hsCRP) as a strong predictor of future cardiovascular (CV) events in primary and secondary prevention.^2^, ^3^, ^4^ However, only in 2017, the ‘proof‐of‐concept’ CANTOS (Canakinumab Anti‐inflammatory Thrombosis Outcomes Study)^5^ Trial was able to demonstrate for the first time that inhibition of the inflammatory cascade resulted in a reduction of future CV events in patients with previous myocardial infarction (MI). Thus, low grade systemic inflammation became a new promising target for intervention to reduce residual CV risk. It took another 7 years until the recent ESC guidelines on the management of chronic coronary syndrome (CCS) recommended that anti‐inflammatory therapy (low dose colchicine 0.5 mg daily) should be considered in all CCS patients with atherosclerotic cardiovascular disease (ASCVD) and an inflammatory burden for risk reduction.^6^ However, are we ready to initiate anti‐inflammatory therapy as widely as we initiate lipid‐lowering medication? Or, even more importantly—should we screen for elevated hsCRP on a routine basis? If not yet implemented, we definitely should do it, since up to 60% of ASCVD patients might be at residual inflammatory risk (RIR) defined by hsCRP values ≥2 mg/L, as impressively shown in a series of investigations worldwide, including for the first time real world data from Sweden.^7^, ^8^, ^9^ Thus, RIR still represents a substantial unmet clinical need.

The present review summarizes our current knowledge on the role and currently available strategies to modulate an inflammatory process in ASCVD patients only (not touching upon other inflammatory conditions such as pericarditis) and discusses the next diagnostic and therapeutic frontiers in targeting RIR.

## 2 RECENT EPIDEMIOLOGICAL EVIDENCE

Over the last 30 years, vascular biology has provided unequivocal evidence that inflammatory processes are involved in all stages of the atherosclerotic process from plaque initiation through plaque growth until the occurrence of ischemic events due to plaque rupture/fissuring or potentially erosion. Therefore, it is not surprising that numerous large‐scale epidemiological and clinical studies have clearly demonstrated that elevated systemic inflammatory biomarkers, such as interleukin‐6 (IL‐6) or hsCRP, have been found to independently predict future events not only in the primary prevention setting but also in those in the acute setting (ACS) or in CCS.^2^, ^3^, ^4^, ^8^, ^9^, ^10^, ^11^, ^12^, ^13^, ^14^, ^15^, ^16^, ^17^, ^18^ However, in contrast to IL‐6, hsCRP has been shown not to be causally involved in atherogenesis by Mendelian Randomization studies.^19^, ^20^ Nonetheless, due to its broad availability, easiness in determination and established cut‐offs for elevated risk, hsCRP has been widely introduced in the clinical routine as a solid marker for the determination of an inflammatory state and for risk stratification. Interestingly, there are several lines of evidence that hsCRP is at least as strong a predictor of major adverse coronary events (MACE) or even mortality as traditional lipoproteins in both primary and secondary prevention. For instance, in a recently published pooled analysis of 31,245 patients at high risk of ASCVD with statin therapy, participating in the PROMINENT (Pemafibrate to Reduce Cardiovascular Outcomes by Reducing Triglycerides in Patients with Diabetes), REDUCE‐IT (Reduction of Cardiovascular Events with Icosapent Ethyl—Intervention Trial) and STRENGTH (Long‐term Outcomes Study to Assess Statin Residual Risk with Epanova in High Cardiovascular Risk Patents with Hypertriglyceridemia) trials, increased hsCRP (top versus bottom quartile) has been strongly associated with incident MACE, CVD mortality, as well as all‐cause mortality with risk estimates (adjusted Hazard ratio (HR)) of 1.31 (95% confidence interval (CI), 1.20–1.43), 2.68 (95% CI, 2.22–3.23) and 2.42 (95% CI, 2.12–2.77), respectively. In contrast, the corresponding HRs for extreme LDL‐C quartiles were 1.07 (95% CI, 0.98–1.17), 1.27 (95% CI, 1.07–1.50) or 1.16 (95% CI, 1.03–1.32), respectively.^21^ Similar results have been seen in 13,970 statin‐intolerant patients participating in the CLEAR‐Outcomes trial (Cholesterol Lowering via Bempedoic Acid, an ACL‐Inhibiting Regimen Outcomes Trial), who were randomly allocated to 180 mg of oral bempedoic acid daily or matching placebo.^22^ The corresponding risk estimates were 1.43 (95% CI, 1.24–1.65) for MACE, 2.00 (95% CI, 1.53–2.61) for CVD mortality and 2.21 (95% CI, 1.79–2.73) for all‐cause mortality for high versus low hsCRP (quartile analysis) and 1.19 (95% CI, 1.04–1.37), 0.90 (95% CI, 0.70–1.17) or 0.95 (95% CI, 0.78–1.16) for LDL‐C quartiles, respectively. Notably, in joint analyses of hsCRP (<2 versus ≥2 mg/L) and LDL‐C (<70 versus ≥70 mg/dL in those on statin therapy or <130 versus ≥130 mg/dL in statin‐intolerant patients) the strong association with outcomes was seen only in the presence of RIR, that is, hsCRP values ≥2 mg/L, irrespective of concomitant LDL‐C concentration. This might indicate that systemic inflammation can be considered an even stronger driver of residual risk than hypercholesterolemia and targeting LDL‐C alone would not completely reduce atherosclerotic risk. In line with this assumption are data from the FOURIER (Further Cardiovascular Outcomes Research With PCSK9 Inhibition in Subjects With Elevated Risk) and SPIRE (Studies of PCSK9 Inhibition and the Reduction in Vascular Events) trials, demonstrating that inflammation can still play a prognostic role even in patients with very low LDL‐C concentration (e.g. median of 30 mg/dL) with a 20% increased risk for the primary CVD endpoint among subjects with hsCRP >3 mg/L compared to those with lower hsCRP concentration (<1 mg/L).^23^, ^24^ More importantly, hypercholesterolemia and inflammation should be considered as a complementary/synergistic condition, especially taking into account a tight interplay of lipoproteins and inflammatory mediators in atherogenesis. Thus, the combined use of intensive lipid‐lowering and anti‐inflammatory therapy might significantly improve CV outcomes and should become standard of care, especially in those with clinical progression despite adequate treatment of traditional CV risk factors. Further indirect evidence supporting a combined approach modifying hypercholesterolemia and systemic inflammation to achieve the strongest benefit in ASCVD risk reduction recently came from the Women's Health Study (WHS) including 27,939 initially healthy U.S. women, who were subsequently followed for 30 years. Women presenting with elevated hsCRP, LDL‐C, as well as an important, genetically determined risk factor, lipoprotein (a) (Lp(a)) in the top quintile showed a strongly increased risk for future CHD events with an adjusted HR of 3.71 (95% CI, 2.94–4.68) compared to participants with no biomarker in the top quintile. Again, if biomarkers were considered separately, hsCRP showed the strongest predictive value for future CVD events, stronger than LDL‐C or Lp(a).^25^

## 3 UNDERLYING PATHOPHYSIOLOGICAL MECHANISMS

The observational evidence for IL‐6 and future ASCVD risk goes hand in hand with what has been reported for hsCRP, which is not surprising since they both are part of the NLRP3 (NOD [nucleotide oligomerization domain], LRR [leucine‐rich repeat] and PYD [pyrin domain]‐containing protein 3) inflammasome pathway. Canonically, a tight interplay between lipoproteins and immune cellular response has been considered a hallmark of atherogenesis, where retained and modified lipoproteins within the arterial wall trigger a local inflammatory process.^1^ Interestingly, the NLRP3 inflammasome might represent a mechanistic link in the lipoprotein–inflammation axis, since various components of cholesterol metabolism represent potent NLRP3 inflammasome inducers, which in turn might lead to the initiation of an innate immune response, a mechanism being currently considered as one of the major contributors of atherogenesis.^26^, ^27^ For instance, oxidized LDLs, abundantly present in atherosclerotic lesions, can mimic damage‐associated molecular pattern molecules (DAMPs) with a subsequent dimerization and activation of pattern recognition receptors on macrophages such as toll‐like receptors (TLR) 2 and TLR 2/4.^26^, ^28^ Furthermore, the generation of intracellular cholesterol crystals with further lysosomal damage, enhanced potassium ion efflux, mitochondrial dysfunction and reactive oxygen species release represents another mechanism of a NLRP3 inflammasome coactivation within macrophages.^29^, ^30^, ^31^ More recently, another important lipid player, namely Lp(a), possessing potent pro‐inflammatory properties and serving as a major carrier of oxidized phospholipids in the circulation,^32^ has been considered as another activator of the NLRP3 inflammasome in monocytes.^33^

**FIGURE 1:**
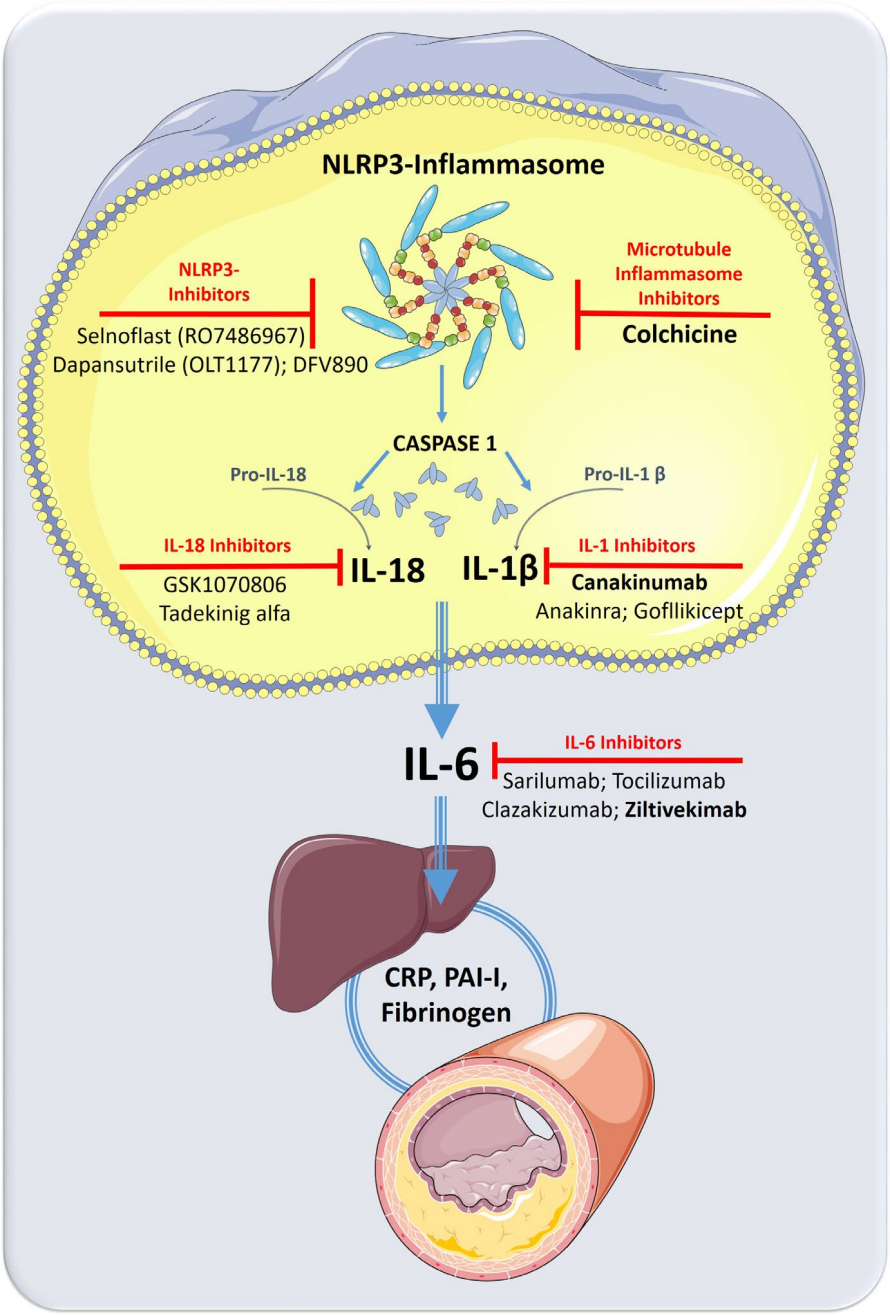
Canonical NLRP3‐inflammasome pathway and potential therapeutic targets in atherosclerotic cardiovascular disease. CRP, C‐reactive protein; IL, interleukin; LRR, leucine‐rich repeat; NLRP3, NOD [nucleotide oligomerisation domain]; PAI‐I, plasminogen‐activator inhibitor‐1; PYD, [pyrin domain]‐containing protein 3. *Source*: Some of the icons of this image are reproduced from Servier Medical Art by Servier under a Creative Commons (CC BY 4.0) licence.

Once activated, the NLRP3 complex stimulates caspase‐1, which in turn results in the release of highly pro‐inflammatory cytokines such as IL‐1β or IL‐18 and subsequently IL‐6 (Figure 1). This finally leads to protein synthesis in the liver with a characteristic shift towards an acute phase pattern with elevated CRP, fibrinogen, serum amyloid A (SAA), plasminogen‐activator inhibitor‐1 (PAI‐1) and others.^34^ The seminal role of the NLRP3 inflammasome and the IL‐1β pathway in atherosclerosis has recently been reviewed in detail.^27^, ^35^

However, experimental studies suggest that activation of the NLRP3 inflammasome requires some aggravating factors (so called ‘second hits’) such as defective cholesterol efflux, hyperglycemia or clonal haematopoiesis.^36^ Indeed, during recent years, clonal haematopoiesis of indeterminate potential (CHIP), being an age‐related phenomenon of cell proliferation/clonal amplification without overt haematological abnormalities, has gained increasing attention as a novel and promising risk determinant of ASCVD.^37^

Among several acquired somatic mutations in leukemogenic genes (in particular e.g. ten eleven translocation 2 (TET2), DNA methyltransferase 3 A (DNMT3A), additional sex combs‐like 1 (ASXL1) or Janus kinase 2 (JAK2)), causing a clonal expansion of haematopoietic cells, TET2 and DNMT3A represent not only the most commonly identified mutated genes associated with CHIP but also mutations, which are mostly related to an accelerated atherosclerosis due to NLRP3 inflammasome activation.^37^, ^38^ For instance, it could be shown that partial bone marrow transplantation with TET2‐deficient cells in mice resulted in a significant increase in atherosclerotic plaque size relative to wild‐type control mice.^39^, ^40^ Of major interest, atherosclerotic plaque growth was highly associated with enhanced IL‐1β/IL‐6 production in mutant mice through overactivation of the NLRP3 inflammasome. Also, in a cohort of approximately 98,000 individuals, an association between TET2 and increased circulating IL‐1β has been demonstrated.^41^ Furthermore, genetic inhibition of the classical IL‐6 signalling pathway due to the commonly occurring *IL6R* p.Asp358Ala coding mutation resulted in a 50% reduction of future ASCVD risk among carriers of CHIP clones, but not in individuals without CHIP, thereby suggesting that subjects with CHIP may be better responders to therapeutic modulation of NLRP3 inflammasome‐driven immunopathology.^42^ Interestingly, more recent investigations suggested that the relationship between CHIP and inflammation might be bidirectional^37^, ^43^ and, for example, pharmacological inhibition of IL‐6 signalling can mitigate *TET2*‐mediated CVD risk in a murine model.^44^

## 4 PHARMACOLOGICAL MODULATION OF INFLAMMATION

### 4.1 The CANTOS trial

Initial evidence that inflammation might be pharmacologically modulated came from large lipid‐lowering trials, which showed a significant reduction of circulating CRP in patients on statin therapy^45^, ^46^, ^47^ with a subsequent significant reduction of future CV events.^48^ The largest risk reduction has been observed among those who achieved reductions of both LDL‐C below 70 mg/dL and hsCRP below 2 mg/L.^47^, ^48^

The first landmark trial, investigating a pharmacological inhibition of the NLRP3 inflammasome‐related pathway, was CANTOS, which tested the hypothesis of whether IL‐1β blockade by a specific monoclonal antibody would reduce the rate of MACE in 10,061 stable post‐MI patients with hsCRP concentrations ≥2 mg/L despite maximally tolerated statin treatment.^5^ A significant reduction in MACE of 15% over a median of 3.7 years has been observed in the Canakinumab group, compared to placebo. Notably, subjects who achieved a hsCRP of less than 2 mg/L at 3 months benefited most from anti‐inflammatory treatment, showing a 25% reduction in MACE.^49^ Of note, a particularly strong relative risk reduction (RRR) of 62% for MACE was seen with canakinumab in exploratory analyses in subjects harbouring TET2 mutations,^50^ again reflecting a strong interplay between NLRP3 inflammasome and CHIP. Despite these promising results, direct targeting of IL‐1β by canakinumab was associated with a small but statistically significant increased risk for fatal infections (0.31 vs. 0.18 events per 100 person‐years; *p* = 0.02).^5^

### 4.2 The CIRT trial

In contrast, results from the large Cardiovascular Inflammation Reduction Trial (CIRT) trial were rather disappointing.^51^ Here, 4786 stable high‐risk patients, either post‐MI or presenting with multivessel disease and diabetes or metabolic syndrome on standard secondary prevention care, were randomly allocated to treatment with low‐dose methotrexate versus placebo. Methotrexate therapy neither decreased MACE over 5 years (HR 1.01 (95% CI, 0.82–1.25) versus placebo) nor did it lower hsCRP.^51^ The latter is not surprising, since the CIRT trial did not require elevated hsCRP at enrollment and the differences in median hsCRP between the methotrexate and canakinumab arms were 1.53 and 4.20 mg/L, respectively. Moreover, in contrast to canakinumab, methotrexate acts via inhibition of aminoimidazole‐4‐carboximaide ribonucleotide with subsequent elevations in adenosine levels and has no specific effects on the IL‐1β/IL‐6 pathway.^52^

### 4.3 Colchicine trials

Another compound, which can directly inhibit the inflammatory pathway targeting the NLRP‐3 inflammasome, is colchicine. Having been known for several decades as a long‐term agent to treat familial Mediterranean fever (FMF)^53^ as well as a first‐line therapy (standard of care) for acute and recurrent pericarditis, colchicine has also been approved in 2023 by the U.S. Food and Drug Administration (FDA) as the first anti‐inflammatory drug to reduce the risk of ASCVD. This remarkable revival of colchicine is based largely on the results of two large outcome trials, showing that low‐dose treatment (0.5 mg daily) resulted in a risk reduction by 20%–30% in both an ACS setting (first 30 days after MI) (COLchicine Cardiovascular Outcomes Trial (COLCOT); *n* = 4755)^54^ as well as among patients with CCS in the LoDoCo2 study (Low‐dose colchicine for secondary prevention of cardiovascular disease 2; *n* = 5522).^55^ Interestingly, the observed reduction in MACE was stronger than that observed for canakinumab. Although both agents are targeted against NLRP3 inflammasome sequelae, colchicine, in contrast to canakinumab, which selectively targets IL‐1β, leaving IL‐1α untouched, has much broader effects on inflammation, predominantly as a consequence of the inhibition of tubulin polymerization.^56^ Therefore, it is not surprising that colchicine treatment reduces not only NLRP3 inflammasome‐related cytokines, but also other important mediators, such as myeloperoxidase.^57^ Moreover, a further 23 anti‐inflammatory/atherogenic biomarkers, such as fibroblast growth factor or insulin‐like growth factor‐binding protein, are also upregulated upon colchicine therapy.^57^ Interestingly, recent data investigating the role of colchicine on Lp(a)‐ and oxidized lipoprotein‐associated risk in a LoDoCo2 biomarker subpopulation have revealed that treatment with colchicine reduced ASCVD independently of the accompanying Lp(a) concentration.^58^ However, in those with elevated Lp(a) values, the absolute benefits appeared to be higher in those with Lp(a) ≥125 nmol/L, thereby suggesting that colchicine may be more efficacious in subjects with inflammation driven by oxidized apoB‐containing lipoproteins.^58^

The potential benefit of colchicine therapy in ACS patients is still discussed controversially. For instance, preprocedural (during acute or chronic PCI) administration of a much higher colchicine dose of 1.8 mg among 400 subjects of the COLCHICINE‐PCI study did not reduce PCI‐related myocardial injury/MI or MACE neither within 30 days nor 3 years later, despite the prevention of an acute rise in IL‐6 and hsCRP by colchicine.^59^, ^60^ Also in the COPS Trial, including 795 ACS patients with colchicine initiation during the index hospitalization (0.5 mg twice daily in the first month, followed by 0.5 mg daily for the remaining 11 months), no significant differences in the primary endpoint between the studied groups were revealed over a 12‐month follow‐up period, although a trend was seen for the colchicine group (6.1% versus 9.5% in the placebo group; *p* = 0.09).^61^ However, at the 24‐month follow‐up, a significant reduction in the primary endpoint in the colchicine group compared with placebo could be demonstrated (8.1% vs. 13.5%, respectively, *p* = 0.02).^62^ More importantly, these observations would rather suggest the possibility of a legacy effect of colchicine treatment, since the drug was administered only for 12 months.

Of particular interest are the recently published results of the CLEAR SYNERGY (Colchicine and Spironolactone in Patients with Myocardial Infarction/SYNERGY Stent Registry) trial, where colchicine and spironolactone were randomized in a 2‐by‐2 factorial design against placebo in 7062 patients with MI.^63^, ^64^ Treatment with colchicine did not reduce the incidence of the composite primary outcome (CVD death, recurrent MI, stroke or unplanned ischemia‐driven coronary revascularization) over a median follow‐up period of 3 years (HR 0.99 (95% CI, 0.85–1.16)), despite significant hsCRP reduction in the colchicine arm.^63^ However, this study has several limitations, which might have significantly influenced the abovementioned results. First, the colchicine dosage was initially weight‐based, and those with body weight ≥70 kg received twice‐a‐day dosing for the first 3 months, showing a higher than expected discontinuation rate for colchicine during the trial. Furthermore, MI might have been underreported during the COVID‐19 pandemic since an inverted relationship between the incidence of nonfatal MI and all‐cause deaths was found in CLEAR SYNERGY with a MI/all‐cause death ratio of 0.61 (rates of MI and death were 3.1% and 5.1% for a median follow‐up of 3 years). Such a phenomenon was already reported for several trials conducted during the COVID‐19 phase.^65^ Interestingly, a 22% reduction in MACE under colchicine therapy was seen compared to placebo before the pandemic, which is in line with data from COLCOT^54^ and LoDoCo2.^55^ However, these differences were completely lost during the pandemic. Of note, the neutral results of CLEAR‐SYNERGY for spironolactone are also unexpected^64^ and differ from other so far published landmark studies, showing significant benefits of mineralocorticoid receptor antagonists on CV endpoints.^66^, ^67^ Taken together, a possible interference of the COVID‐19 pandemic could not be excluded completely, making results of the CLEAR‐SYNERGY Study difficult to interpret.

At the same time, a recently published collaborative meta‐analysis of six randomized trials comparing colchicine therapy versus placebo/no colchicine among 14,934 patients with prior stroke or prevalent CHD demonstrated a significantly reduced risk of MACE as well as of ischemic stroke by 27% (RR 0.73 (95% CI, 0.65–0.81) for MACE and of 0.73 (95% CI, 0.58–0.90) for ischemic stroke) with similar efficacy among key subgroups (sex, age, diabetes and statin treatment).^68^ This meta‐analysis has two major findings/implications. First, it clearly showed that colchicine is as effective for ischemic stroke reduction as for MACE reduction, thereby arguing the recently completed COlchicine for preveNtion of Vascular Inflammation in Non‐CardioEmbolic stroke (CONVINCE) trial of 3144 patients with ischemic stroke or transient ischemic attack, where colchicine failed to prevent recurrent adverse events.^69^ Although in the CONVINCE trial, numerically fewer recurrent stroke and coronary events were seen in the colchicine group compared to placebo (HR 0.84 (95% CI, 0.68–1.05)), such differences were not statistically significant between both treatment groups. It should be noted here that the CONVINCE trial was prematurely terminated due to budget constraints during the COVID‐19 pandemic before the anticipated number of outcomes was accrued.^69^ Thus, the present meta‐analysis erased some uncertainties about the efficacy of colchicine for secondary prevention of stroke. The second important observation from the Colchicine Cardiovascular Trialists Collaboration was related to the safety of colchicine therapy. Here, no significant differences were found for all‐cause mortality (RR 1.09 (95% CI, 0.89–1.33)), CV death (RR 0.89 (95% CI, 0.65–1.23)) but a signal for noncardiovascular mortality (RR 1.26 (95% CI, 0.97–1.64)), which, however, was not statistically significant and was driven mainly by the LoDoCo2 and COPS studies.^55^, ^61^ Furthermore, the current meta‐analysis showed no increase in specific major causes of death (hospitalizations for new cancer, pneumonia or gastro‐intestinal events).^68^ A small statistically nonsignificant increase in noncardiovascular deaths in those on colchicine therapy initially has raised concerns about its safety, leading to the intensive debates seen during previous years.^70^, ^71^ This evidence came primarily from the LoDoCo‐2 trial, with an increased total and noncardiovascular mortality within the colchicine group.^55^ HRs were found to be 1.21 (95% CI, 0.86–1.71) and 1.51 (95% CI, 0.99–2.31), respectively. However, in a detailed analysis of the causes of death in LoDoCo2, recently published by Opstal et al.^72^ no differences in the incidence of specific causes of death were found in those assigned to colchicine or placebo, being 0.9% versus 0.8% for cancer, 0.3% versus 0.1% for end‐stage pulmonary disease and 0.1% versus 0.1% for infection, respectively. More importantly, age >65 years was the only independent baseline characteristic associated with noncardiovascular death (HR 3.65; 95% CI, 2.06–6.47) in multivariable analysis. Regarding the COPS trial, the observed differences for noncardiovascular death between colchicine and placebo were statistically significant (5 vs. 0; *p* = 0.023).^61^ However, three out of five subjects who died from noncardiovascular causes were not taking colchicine at the time of their death due to early (within first 30 days) discontinuation of colchicine, and one death occurred due to cancer. More importantly, in the 2‐year follow‐up of the study, no difference in the risk of all‐cause or noncardiovascular death between colchicine and placebo was observed.^62^

Another interesting observation has been recently published.^73^ Ou et al. performed a systematic review and network meta‐analysis of 17 randomized controlled trials, including 85,823 statin‐treated participants with ASCVD, who additionally were on colchicine, PCSK9 inhibitor, ezetimibe or bempedoic acid therapies. Compared to statin treatment alone, further reduction of MACE was seen for colchicine, PCSK9 inhibitor and ezetimibe. Interestingly, the strongest beneficial effects were found for colchicine (RR 0.67; 95% CI, 0.59–0.77), while a 15% reduction in MACE was found for PCSK9 inhibition (RR 0.85; 95% CI, 0.81–0.90), and 7% for ezetimibe (RR 0.93; 95% CI 0.89–0.98) (all compared with statin treatment alone).^73^ However, an increased risk of noncardiovascular death was also seen in patients on colchicine‐statin combination therapy, compared with statin treatment alone (RR 1.45; 95% CI 1.04–2.03), again probably mainly driven by the LoDoCo2 and COPS trials,^55^, ^61^ as discussed above.

Since low‐dose colchicine (0.5 mg once daily) for secondary prevention of ASCVD is intended to be used long‐term, the existing experience with colchicine among patients with FMF^53^ would rather speak for the stable safety profile of this drug. Nonetheless, further studies with long‐term follow‐up in future randomized trials as well as real‐world data are urgently needed to ensure a safe use of colchicine in the secondary prevention of ASCVD. Until then, ongoing colchicine therapy requires regular monitoring of blood counts for cytopenia development and if renal function for eGFR falls <45 mL/min/1.73 m^2^, both conditions which would represent strong reasons to stop treatment.^71^

Major randomized clinical CV outcome trials on anti‐inflammatory therapy in ASCVD are summarized in Table 1.

**TABLE 1:** Anti‐inflammatory CVOTs in ASCVD.

Trial	Author, year	Study population	Median FU	Treatment/comparator	Main outcome
Canakinumab					
CANTOS	Ridker et al. 2017	10,061 stable post‐MI patients	3.7 yrs	Canakinumab 150 mg s.c. 1× 3 mo/placebo	MACE: HR 0.85 (95% CI, 0.74–0.98)
Methotrexate					
CIRT	Ridker et al. 2018	4786 patients with previous MI or multivessel CHD	2.3 yrs	Methotrexate 15 mg, once weekly/placebo	MACE: HR 0.96 (95% CI, 0.79–1.16)
Colchicine					
LoDoCo	Nidorf et al. 2013	532 patients with clinically stable (>6 months) CHD	3 yrs	0.5 mg once daily/no colchicine	MACE: HR 0.33 (95% CI, 0.18–0.59)
COLCOT	Tardif et al. 2019	4745 post MI patients	22.6 mo	0.5 mg once daily/placebo	MACE: HR 0.77 (95% CI, 0.61–0.96)
LoDoCo2	Nidorf et al. 2020	5522 patients with clinically stable (>6 months) chronic CHD	28.6 mo	0.5 mg once daily/placebo	MACE: HR 0.72 (95% CI, 0.57–0.92)
COPS	Tong et al. 2020	795 patients with ACS and presence of CHD	12 mo	0.5 mg twice daily for 1 month, followed by .5 mg once daily/placebo	Reduction in MACE: 6.1% vs. 9.5%; *p* = 0.09
	Tong et al. 2021		24 mo		Reduction in MACE: 8.1% vs. 13.5%; *p* = 0.02
CONVINCE	Kelly et al. 2024	3144 patients with non‐severe ischemic stroke or high‐risk TIA	33.6 mo	0.5 mg once daily/no colchicine	MACE HR 0.84 (95% CI, 0.68–1.05)
Meta‐analysis	Fiolet et al. 2024	14,934 patients with prior stroke or CHD	NA	NA	Ischemic stroke: RR 0.73 (95% CI, 0.58–0.90) MACE: RR 0.73 (95% CI, 0.65–0.81)
CLEAR SYNERGY	Jolly et al. 2024	7062 patients with ACS	3 yrs	First 90 days—weight dependent; after 0.5 mg once daily/placebo	MACE: HR 0.99 (95% CI, 0.85–1.16)

Abbreviations: ACS, acurecoronary syndrome; CANTOS, canakinumab anti‐inflammatory thrombosis outcomes study; CHD, coronary heart disease; CI, confidence interval; CIRT, cardiovascular inflammation reduction trial; CLEAR SYNERGY, Colchicine and Spironolactone in Patients with Myocardial Infarction/SYNERGY Stent Registry; COLCOT, colchicine cardiovascular outcomes trial; CONVINCE, COlchicine for preveNtion of Vascular Inflammation in Non‐CardioEmbolic stroke; CVOTs, cardiovascular outcome trials; HR, hazard ratio; LoDoCo2, low dose colchicine for secondary prevention of cardiovascular disease 2; MACE, major adverse cardiac event; MI, myocardial infarction; mo, months; NA, not applicable; TIA, transient ischemic attack; vs., versus; yrs., years.

## 5 NEW FRONTIERS

Unfortunately, despite such promising treatment strategies, there are still some emerging issues that have to be overcome for more successful combat of inflammation in ASCVD. For instance, it is well known that patients on canakinumab are still at substantial residual inflammatory risk, as it has been shown by a secondary CANTOS analysis.^74^ Each tertile increase of both IL‐6 and IL‐18 concentrations, measured 3 months after canakinumab initiation, was still associated with a higher risk for MACE (42% for IL‐6 and 15% for IL‐18), thereby suggesting that even more profound inhibition of the inflammatory pathway affecting upstream NLRP3‐inflammasome is needed to reduce RIR more effectively. On the other hand, potential risks due to stronger interaction with immune homeostasis should not be underestimated, since even canakinumab's use was associated with a small but statistically significant risk for fatal infections (0.31 vs. 0.18 events per 100 person‐years; *p* = 0.02). In addition, treatment with colchicine has also several limitations, especially due to its contraindication in patients with impaired renal function.^71^ Thus, new targets, such as, for example, direct inhibition of IL‐6 as a downstream molecule of the NLRP3 inflammasome, might represent another promising option.

To date there are several IL‐6 pathway inhibitors under investigation. For instance, the administration of tocilizumab (monoclonal antibody against IL‐6) after MI was able to reduce myocardial/reperfusion injuries.^75^ Furthermore, the application of ziltivekimab, a human monoclonal antibody targeting IL‐6 in high‐risk CV patients with chronic kidney disease and an increased inflammatory state in the phase‐2 RESCUE trial (Trial to Evaluate Reduction in Inflammation in Patients With Advanced Chronic Renal Disease Utilizing Antibody Mediated IL‐6 Inhibition) resulted in significant reductions of several pro‐inflammatory and prothrombotic biomarkers, including an over 90% reduction in hsCRP at the highest dose tested.^76^

Following the RESCUE trial, several large randomized phase 3 outcome trials are now being conducted, investigating the effect of ziltivekimab on atherosclerotic events in the setting of chronic kidney disease (ZEUS trial: the effects of ziltivekimab vs. placebo on cardiovascular outcomes in participants with established atherosclerotic cardiovascular disease, chronic kidney disease and systemic inflammation; NCT05021835), in heart failure with preserved ejection fraction (HERMES trial: effects of ziltivekimab vs. placebo on morbidity and mortality in patients with heart failure with mildly reduced or preserved ejection fraction and systemic inflammation; NCT05636176) and in individuals in the acute phase of MI (ARTEMIS trial: effects of ziltivekimab vs. placebo on cardiovascular outcomes in patients with acute myocardial infarction; NCT06118281).

In addition to IL‐6 inhibition, there are several other interesting compounds, such as the IL‐1 receptor antagonist anakinra, as a short plasma half‐life recombinant nonglycosylated protein, blocking both IL‐1α and IL‐1β, or goflikicept (RPH‐104), being a fusion protein, functioning as a trap for both IL‐1 isoforms (IL‐1α and IL‐1β), which are currently under investigation in phase II trials in subjects with atherosclerotic or pericardial diseases.^34^, ^77^, ^78^, ^79^ For the comprehensive overview of novel anti‐inflammatory targets as well as upcoming clinical trial please see the recent review by Potere et al.^80^

**FIGURE 2:**
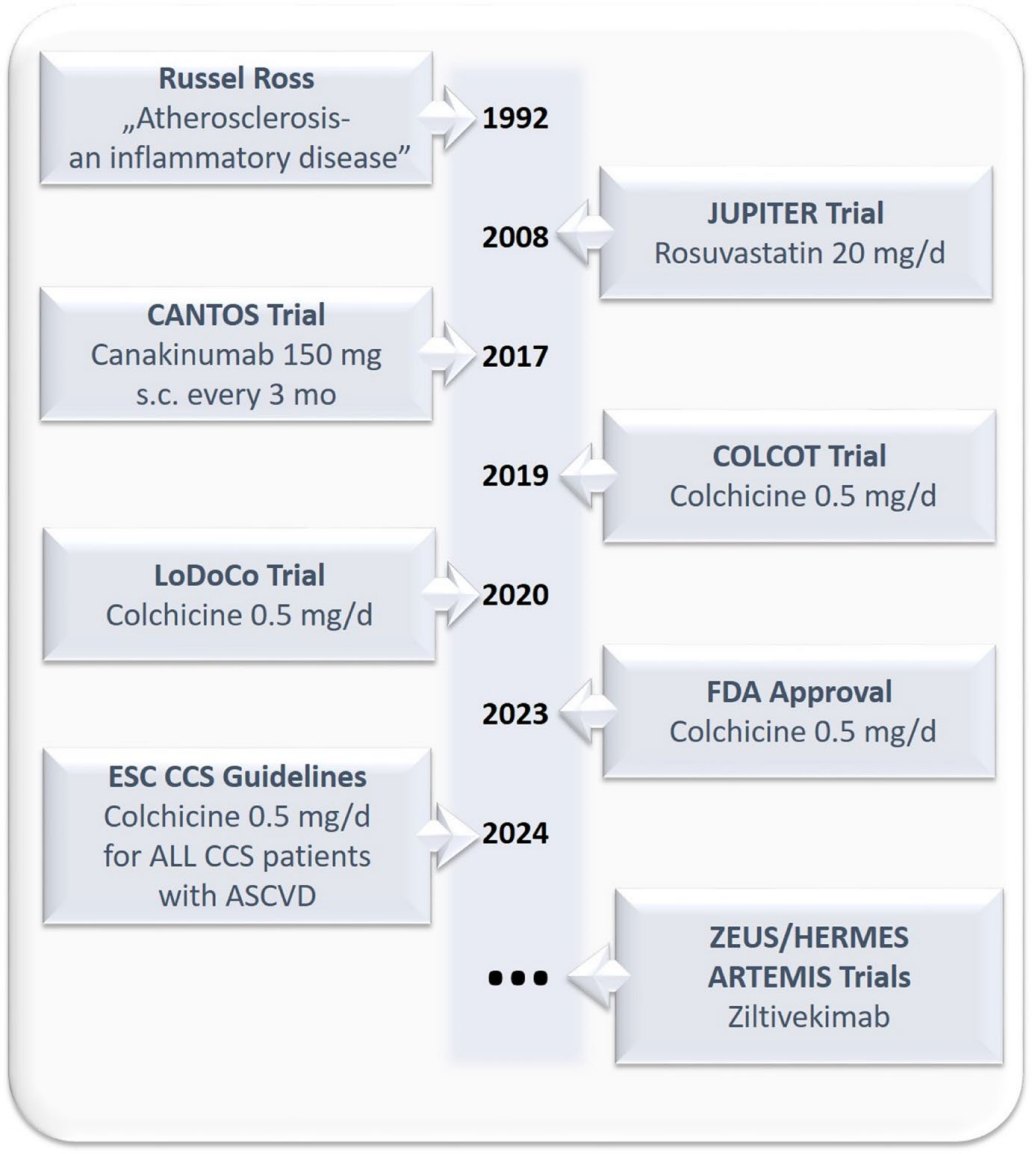
Key milestones of anti‐inflammatory therapy in atherosclerotic cardiovascular disease. ARTEMIS, effects of ziltivekimab versus placebo on cardiovascular outcomes in patients with acute myocardial infarction; ASCVD, atherosclerotic cardiovascular disease; CANTOS, canakinumab anti‐inflammatory thrombosis outcomes study; CCS, chronic coronary syndrome; COLCOT, colchicine cardiovascular outcomes trial; ESC, European Society of Cardiology; FDA, food and drug administration; JUPITER, justification for the use of statins in prevention: an intervention trial evaluating rosuvastatin; LoDoCo2, low dose colchicine for secondary prevention of cardiovascular disease 2; RIR, residual inflammatory risk; ZEUS, the effects of ziltivekimab versus placebo on cardiovascular outcomes in participants with established atherosclerotic cardiovascular disease, chronic kidney disease and systemic inflammation; HERMES, effects of ziltivekimab versus placebo on morbidity and mortality in patients with heart failure with mildly reduced or preserved ejection fraction and systemic inflammation.

Thus, targeting inflammation offers a unique possibility to address residual CV risk in patients with ASCVD. Figure 2 shows the key milestones of anti‐inflammatory therapy in atherosclerosis.

## 6 NONINVASIVE DETECTION OF VASCULAR INFLAMMATION: PERICORONARY FAT ATTENUATION

Apart from systemic inflammation, which is usually measured in the circulation by various inflammatory markers, measuring inflammatory activity within the coronary vessels may be another interesting approach to improve risk prediction and to guide the management of patients with RIR, especially considering that systemic inflammation is not specific enough to reflect what is going on in the vessel wall. Indeed, it is well known that inflammation within the coronary endothelium results in a vulnerable plaque phenotype^81^ and timely identification of unstable plaque whose imminent rupture will cause ACS would be a situation, that every cardiologist would like to see. Although molecular correlates of vulnerable plaque are detectable by several imaging methods such as intravascular ultrasound or optical coherence tomography,^82^ these modalities are still invasive. Recently, a novel noninvasive imaging biomarker of plaque instability has been introduced, which might identify inflamed coronary vessels directly by using perivascular fat phenotyping.^83^ This new tool to monitor atherosclerotic plaque ‘activity’, named perivascular ‘fat attenuation index’ or FAI might be crucial in identifying high risk vulnerable plaques that are prone to rupture, with higher FAI being indicative of a greater degree of vascular inflammation.^84^ The whole FAI concept is based on the fact that inflamed coronary arteries can change the composition of the surrounding pericoronary adipose tissue (PCAT) by secretion of proinflammatory mediators.^85^ This, in turn, promotes lipolysis of fat deposits within adipocytes with subsequent decrease in lipid content and promotion of water storage. Such a shift from a lipid to a more aqueous phase of adipose tissue can be captured by high‐resolution coronary computed tomography angiography (CCTA) as increased attenuation. This approach might therefore identify both inflamed coronary arteries without atherosclerosis and vulnerable atherosclerotic plaques prone to rupture, thereby representing a novel and promising imaging biomarker of vascular inflammation.^84^

The clinical performance of FAI has been extensively evaluated and summarized in a series of comprehensive reviews, including a clinical consensus statement from the European Society of Cardiology Working Group on Coronary Pathophysiology and Microcirculation.^84^, ^85^, ^86^, ^87^ More importantly, two studies, the CRISP‐CT (Cardiovascular RISk Prediction using Computed Tomography) study as well as the recently published ORFAN (Oxford Risk Factors And Non‐invasive imaging) study, have evaluated whether coronary inflammation assessed by perivascular fat phenotyping might predict future coronary events independently of the calcium score.^88^, ^89^ The first study, CRISP‐CT, was a post hoc analysis of prospectively obtained outcome data from two independent clinical cohorts and included about 4000 patients with clinically indicated CCTA.^88^ It could be shown that the presence of increased inflammatory burden in the coronary arteries, as assessed by FAI, was associated with both all‐cause and CV mortality, and this association was independent of traditional risk factors and various CCTA metrics. The second study, the ORFAN Study, has included 40,091 patients from seven UK hospitals who underwent clinically indicated CCTA and who were followed for approximately 7 years.^89^ One of the most intriguing findings of the ORFAN Study was that 81.1% of all patients who had a clinical indication for CCTA were without obstructive coronary artery disease (CAD). Moreover, over a median follow‐up of 2.7 years, there were almost twice as many events in the group without obstructive CAD compared to those with obstruction. Within the same analysis, the authors have further integrated another cohort of 3393 stable CAD patients with a longer follow‐up period than within the entire population. Impressively, those who were within the top quartile of the FAI‐Score of any coronary artery demonstrated a 10‐ to 20‐times higher cardiac mortality risk and a 7‐to 9‐times higher risk for MACE, compared to those with lower inflammation, that is, being in the bottom quartile of the FAI score.^89^ Even in those with nonobstructive CAD, having a high FAI was associated with a greater risk of MACE (HR 4.77; 95% CI, 3.40–6.69) and cardiac mortality (HR 10.49; 95% CI 5.25–20.95) than in those having lower FAI. More importantly, the number of inflamed arteries was associated with outcome in an additive manner: having all three coronary vessels inflamed was associated with a 29‐fold increased risk for cardiac mortality (corresponding HR of 28.9 (95% CI, 13.9–63.9)) and a 12‐fold increased risk for MACE (HR 12.6 (95% CI, 8.52–18.61)) over a median follow‐up of 7.7 years, compared to those with no inflamed arteries, in both the presence and absence of obstructive CAD.

Thus, FAI seems to be a very promising research tool to integrate perivascular fat inflammation into personalized cardiovascular risk stratification. Importantly, several standardization efforts have already been undertaken.^90^ Nevertheless, several important issues remain to be addressed, such as the interplay between FAI score and circulating biomarkers such as hsCRP or IL‐6, patient selection strategies or the lack of large randomized clinical trials to reduce coronary inflammation.

## 7 CONCLUSION

Targeting inflammation offers a unique possibility to address the residual inflammatory risk encountered in individuals with well‐controlled lipid profiles. However, despite FDA approval and guideline recommendations from the European Society of Cardiology, the implementation of low‐dose colchicine for the secondary prevention of ASCVD is still negligible. More importantly, we have enough evidence that the size of the population potentially benefitting from targeted anti‐inflammatory strategies for secondary ASCVD prevention is much larger than initially assumed, since every three out of five ASCVD patients might have clinically relevant low‐grade systemic inflammation, indicated by a validated threshold for risk assessment of hsCRP of ≥2 mg/L.^9^ Therefore, collaborative efforts for promotion and education programs aimed at a broad adoption of anti‐inflammatory therapy in clinical practice (probably in form of a consensus statement from the leading cardiac societies worldwide) are urgently needed to overcome physician inertia and to begin a simple universal screening for elevated hsCRP on a routine basis in the secondary ASCVD prevention. That might be a first step and act as a gatekeeper to solve the problem of targeting inflammatory burden which is under‐diagnosed and under‐treated to date.

## CONFLICT OF INTEREST STATEMENT

NA reports receiving consulting and lecture fees from Novartis, a lecture fee from Amgen and grant support from Novartis. WK reports receiving consulting fees and lecture fees from AstraZeneca, Novartis, Daiichi‐Sankyo, LIB Therapeutics and Amgen, consulting fees from Pfizer, the Medicines Company, DalCor Pharmaceuticals, Kowa, Corvidia, Genentech, Esperion, Novo Nordisk, OMEICOS, TenSixteen Bio and New Amsterdam Pharma, lecture fees from Novartis, Berlin‐Chemie, Bristol‐Myers Squibb and Sanofi, and grant support and provision of reagents from Singulex, Abbott, Roche Diagnostics and Dr. Beckmann Pharma; WK has been a member of the executive steering committees of JUPITER, CANTOS, SPIRE, GLAGOV and COLCOT.

## References

[1] Ross R . Atherosclerosis‐‐an inflammatory disease. N Engl J Med. 1999;340:115‐126. 9887164 10.1056/NEJM199901143400207

[2] Liuzzo G , Biasucci LM , Gallimore JR , et al. The prognostic value of C‐reactive protein and serum amyloid a protein in severe unstable angina. N Engl J Med. 1994;331:417‐424. 7880233 10.1056/NEJM199408183310701

[3] Ridker PM , Cushman M , Stampfer MJ , Tracy RP , Hennekens CH . Inflammation, aspirin, and the risk of cardiovascular disease in apparently healthy men. N Engl J Med. 1997;336:973‐979. 9077376 10.1056/NEJM199704033361401

[4] Koenig W , Sund M , Fröhlich M , et al. C‐reactive protein, a sensitive marker of inflammation, predicts future risk of coronary heart disease in initially healthy middle‐aged men: results from the MONICA (monitoring trends and determinants in cardiovascular disease) Augsburg cohort study, 1984 to 1992. Circulation. 1999;99:237‐242. 9892589 10.1161/01.cir.99.2.237

[5] Ridker PM , Everett BM , Thuren T , et al. Anti‐inflammatory therapy with canakinumab for atherosclerotic disease. N Engl J Med. 2017;377:1119‐1131. 28845751 10.1056/NEJMoa1707914

[6] Vrints C , Andreotti F , Koskinas KC , et al. 2024 ESC guidelines for the management of chronic coronary syndromes. Eur Heart J. 2024;45:3415‐3537. 39210710 10.1093/eurheartj/ehae177

[7] Nanna M , Sloan L , Faurby M , et al. Abstract 11398: prevalence and characteristics of systemic inflammation in adults with atherosclerotic cardiovascular disease and chronic kidney disease: results from the national health and nutrition examination survey. Circulation. 2022;146(Suppl_1):A11398. doi:10.1161/circ.146.suppl_1.11398

[8] Burger PM , Pradhan AD , Dorresteijn JAN , et al. C‐reactive protein and risk of cardiovascular events and mortality in patients with various cardiovascular disease locations. Am J Cardiol. 2023;197:13‐23. 37218417 10.1016/j.amjcard.2023.03.025

[9] Mazhar F , Faucon AL , Fu EL , et al. Systemic inflammation and health outcomes in patients receiving treatment for atherosclerotic cardiovascular disease. Eur Heart J. 2024;45:4719‐4730. 39211962 10.1093/eurheartj/ehae557 PMC11578643

[10] Ridker PM , Hennekens CH , Buring JE , Rifai N . C‐reactive protein and other markers of inflammation in the prediction of cardiovascular disease in women. N Engl J Med. 2000;342:836‐843. 10733371 10.1056/NEJM200003233421202

[11] Cushman M , Arnold AM , Psaty BM , et al. C‐reactive protein and the 10‐year incidence of coronary heart disease in older men and women: the cardiovascular health study. Circulation. 2005;112(1):25‐31. doi:10.1161/CIRCULATIONAHA.104.504159 15983251

[12] Lindahl B , Toss H , Siegbahn A , Venge P , Wallentin L . Markers of myocardial damage and inflammation in relation to long‐term mortality in unstable coronary artery disease. FRISC study group. Fragmin during instability in coronary artery disease. N Engl J Med. 2000;343:1139‐1147. 11036119 10.1056/NEJM200010193431602

[13] Kaptoge S , Seshasai SR , Gao P , et al. Inflammatory cytokines and risk of coronary heart disease: new prospective study and updated meta‐analysis. Eur Heart J. 2014;35:578‐589. 24026779 10.1093/eurheartj/eht367 PMC3938862

[14] Ridker PM , Rifai N , Stampfer MJ , Hennekens CH . Plasma concentration of interleukin‐6 and the risk of future myocardial infarction among apparently healthy men. Circulation. 2000;101:1767‐1772. 10769275 10.1161/01.cir.101.15.1767

[15] Held C , White HD , Stewart RAH , et al. Inflammatory biomarkers Interleukin‐6 and C‐reactive protein and outcomes in stable coronary heart disease: experiences from the STABILITY (stabilization of atherosclerotic plaque by initiation of Darapladib therapy) Trial. J Am Heart Assoc. 2017;6:e005077. 29066452 10.1161/JAHA.116.005077 PMC5721818

[16] Fanola CL , Morrow DA , Cannon CP , et al. Interleukin‐6 and the risk of adverse outcomes in patients after an acute coronary syndrome: observations from the SOLID‐TIMI 52 (stabilization of plaque using Darapladib‐thrombolysis in myocardial infarction 52) trial. J Am Heart Assoc. 2017;6(10):e005637. doi:10.1161/JAHA.117.005637 29066436 PMC5721825

[17] Ridker PM , MacFadyen JG , Glynn RJ , Bradwin G , Hasan AA , Rifai N . Comparison of interleukin‐6, C‐reactive protein, and low‐density lipoprotein cholesterol as biomarkers of residual risk in contemporary practice: secondary analyses from the cardiovascular inflammation reduction trial. Eur Heart J. 2020;41:2952‐2961. 32221587 10.1093/eurheartj/ehaa160 PMC7453833

[18] Khan MS , Talha KM , Maqsood MH , et al. Interleukin‐6 and cardiovascular events in healthy adults: MESA. JACC Adv. 2024;3:101063. 39077632 10.1016/j.jacadv.2024.101063 PMC11284704

[19] IL6R Genetics Consortium Emerging Risk Factors Collaboration , Sarwar N , Butterworth AS , et al. Interleukin‐6 receptor pathways in coronary heart disease: a collaborative meta‐analysis of 82 studies. Lancet. 2012;379(9822):1205‐1213. doi:10.1016/S0140-6736(11)61931-4 22421339 PMC3316940

[20] Dehghan A , Dupuis J , Barbalic M , et al. Meta‐analysis of genome‐wide association studies in >80 000 subjects identifies multiple loci for C‐reactive protein levels. Circulation. 2011;123:731‐738. 21300955 10.1161/CIRCULATIONAHA.110.948570 PMC3147232

[21] Ridker PM , Bhatt DL , Pradhan AD , et al. Inflammation and cholesterol as predictors of cardiovascular events among patients receiving statin therapy: a collaborative analysis of three randomised trials. Lancet. 2023;401:1293‐1301. 36893777 10.1016/S0140-6736(23)00215-5

[22] Ridker PM , Lei L , Louie MJ , et al. Inflammation and cholesterol as predictors of cardiovascular events among 13,970 contemporary high‐risk patients with statin intolerance. Circulation. 2024;149:28‐35. 37929602 10.1161/CIRCULATIONAHA.123.066213 PMC10752259

[23] Bohula EA , Giugliano RP , Leiter LA , et al. Inflammatory and cholesterol risk in the FOURIER trial. Circulation. 2018;138(2):131‐140. doi:10.1161/CIRCULATIONAHA.118.034032 29530884

[24] Pradhan AD , Aday AW , Rose LM , Ridker PM . Residual inflammatory risk on treatment with PCSK9 inhibition and statin therapy. Circulation. 2018;138:141‐149. 29716940 10.1161/CIRCULATIONAHA.118.034645 PMC8108606

[25] Ridker PM , Moorthy MV , Cook NR , Rifai N , Lee IM , Buring JE . Inflammation, cholesterol, lipoprotein(a), and 30‐year cardiovascular outcomes in women. N Engl J Med. 2024;391:2087‐2097. 39216091 10.1056/NEJMoa2405182 PMC11711015

[26] Gong T , Liu L , Jiang W , Zhou R . DAMP‐sensing receptors in sterile inflammation and inflammatory diseases. Nat Rev Immunol. 2020;20:95‐112. 31558839 10.1038/s41577-019-0215-7

[27] Toldo S , Abbate A . The role of the NLRP3 inflammasome and pyroptosis in cardiovascular diseases. Nat Rev Cardiol. 2024;21:219‐237. 37923829 10.1038/s41569-023-00946-3 PMC11550901

[28] Miller YI , Choi SH , Wiesner P , et al. Oxidation‐specific epitopes are danger‐associated molecular patterns recognized by pattern recognition receptors of innate immunity. Circ Res. 2011;108:235‐248. 21252151 10.1161/CIRCRESAHA.110.223875 PMC3075542

[29] Duewell P , Kono H , Rayner KJ , et al. NLRP3 inflammasomes are required for atherogenesis and activated by cholesterol crystals. Nature. 2010;464(7293):1357‐1361. doi:10.1038/nature08938 20428172 PMC2946640

[30] Kasikara C , Doran AC , Cai B , Tabas I . The role of non‐resolving inflammation in atherosclerosis. J Clin Invest. 2018;128:2713‐2723. 30108191 10.1172/JCI97950 PMC6025992

[31] Abais JM , Xia M , Zhang Y , Boini KM , Li PL . Redox regulation of NLRP3 inflammasomes: ROS as trigger or effector? Antioxid Redox Signal. 2015;22:1111‐1129. 25330206 10.1089/ars.2014.5994 PMC4403231

[32] Kronenberg F , Mora S , Stroes ESG , et al. Lipoprotein(a) in atherosclerotic cardiovascular disease and aortic stenosis: a European Atherosclerosis Society consensus statement. Eur Heart J. 2022;43:3925‐3946. 36036785 10.1093/eurheartj/ehac361 PMC9639807

[33] Dzobo KE , Cupido AJ , Mol BM , et al. Diacylglycerols and lysophosphatidic acid, enriched on lipoprotein(a), contribute to monocyte inflammation. Arterioscler Thromb Vasc Biol. 2024;44:720‐740. 38269588 10.1161/ATVBAHA.123.319937 PMC10880937

[34] Abbate A , Toldo S , Marchetti C , Kron J , van Tassell BW , Dinarello CA . Interleukin‐1 and the inflammasome as therapeutic targets in cardiovascular disease. Circ Res. 2020;126:1260‐1280. 32324502 10.1161/CIRCRESAHA.120.315937 PMC8760628

[35] Del Buono MG , Bonaventura A , Vecchié A , et al. Pathogenic pathways and therapeutic targets of inflammation in heart diseases: a focus on Interleukin‐1. Eur J Clin Investig. 2024;54:e14110. 37837616 10.1111/eci.14110

[36] Tall AR , Bornfeldt KE . Inflammasomes and atherosclerosis: a mixed picture. Circ Res. 2023;132:1505‐1520. 37228237 10.1161/CIRCRESAHA.123.321637 PMC10213995

[37] Oren O , Small AM , Libby P . Clonal hematopoiesis and atherosclerosis. J Clin Invest. 2024;134:e180066. 39352379 10.1172/JCI180066 PMC11444192

[38] Cobo I , Tanaka T , Glass CK , Yeang C . Clonal hematopoiesis driven by DNMT3A and TET2 mutations: role in monocyte and macrophage biology and atherosclerotic cardiovascular disease. Curr Opin Hematol. 2022;29:1‐7. 34654019 10.1097/MOH.0000000000000688 PMC8639635

[39] Fuster JJ , MacLauchlan S , Zuriaga MA , et al. Clonal hematopoiesis associated with TET2 deficiency accelerates atherosclerosis development in mice. Science. 2017;355(6327):842‐847. doi:10.1126/science.aag1381 28104796 PMC5542057

[40] Jaiswal S , Natarajan P , Silver AJ , et al. Clonal hematopoiesis and risk of atherosclerotic cardiovascular disease. N Engl J Med. 2017;377(2):111‐121. doi:10.1056/NEJMoa1701719 28636844 PMC6717509

[41] Bick AG , Weinstock JS , Nandakumar SK , et al. Inherited causes of clonal haematopoiesis in 97,691 whole genomes. Nature. 2020;586:763‐768. 33057201 10.1038/s41586-020-2819-2 PMC7944936

[42] Bick AG , Pirruccello JP , Griffin GK , et al. Genetic interleukin 6 signaling deficiency attenuates cardiovascular risk in clonal hematopoiesis. Circulation. 2020;141:124‐131. 31707836 10.1161/CIRCULATIONAHA.119.044362 PMC7008855

[43] Uddin MM , Saadatagah S , Niroula A , et al. Long‐term longitudinal analysis of 4,187 participants reveals insights into determinants of clonal hematopoiesis. Nat Commun. 2024;15:7858. 39251642 10.1038/s41467-024-52302-9 PMC11385577

[44] Liu W , Yalcinkaya M , Maestre IF , et al. Blockade of IL‐6 signaling alleviates atherosclerosis in Tet2‐deficient clonal hematopoiesis. Nat Cardiovasc Res. 2023;2:572‐586. 37539077 10.1038/s44161-023-00281-3 PMC10399458

[45] Ridker PM , Rifai N , Pfeffer MA , Sacks F , Braunwald E . Long‐term effects of pravastatin on plasma concentration of C‐reactive protein. Circulation. 1999;100:230‐235. 10411845 10.1161/01.cir.100.3.230

[46] Albert MA , Danielson E , Rifai N , Ridker PM , PRINCE Investigators . Effect of statin therapy on C‐reactive protein levels: the pravastatin inflammation/CRP evaluation (PRINCE): a randomized trial and cohort study. JAMA. 2001;286:64‐70. 11434828 10.1001/jama.286.1.64

[47] Bohula EA , Giugliano RP , Cannon CP , et al. Achievement of dual low‐density lipoprotein cholesterol and high‐sensitivity C‐reactive protein targets more frequent with the addition of ezetimibe to simvastatin and associated with better outcomes in IMPROVE‐IT. Circulation. 2015;132:1224‐1233. 26330412 10.1161/CIRCULATIONAHA.115.018381

[48] Ridker PM , Danielson E , Fonseca FA , et al. Rosuvastatin to prevent vascular events in men and women with elevated C‐reactive protein. N Engl J Med. 2008;359:2195‐2207. 18997196 10.1056/NEJMoa0807646

[49] Ridker PM , MacFadyen JG , Everett BM , et al. Relationship of C‐reactive protein reduction to cardiovascular event reduction following treatment with canakinumab: a secondary analysis from the CANTOS randomized controlled trial. Lancet. 2018;391:319‐328. 29146124 10.1016/S0140-6736(17)32814-3

[50] Svensson EC , Madar A , Campbell CD , et al. TET2‐driven clonal hematopoiesis and response to canakinumab: an exploratory analysis of the CANTOS randomized clinical trial. JAMA Cardiol. 2022;7:521‐528. 35385050 10.1001/jamacardio.2022.0386 PMC8988022

[51] Ridker PM , Everett BM , Pradhan A , et al. Low‐dose methotrexate for the prevention of atherosclerotic events. N Engl J Med. 2019;380:752‐762. 30415610 10.1056/NEJMoa1809798 PMC6587584

[52] Chan ES , Cronstein BN . Methotrexate – how does it really work? Nat Rev Rheumatol. 2010;6:175‐178. 20197777 10.1038/nrrheum.2010.5

[53] Ben‐Chetrit E , Nidorf M , Falk R , Ridker PM . Inflammation, colchicine, and atherosclerotic disease: is familial Mediterranean fever an exception that proves the rule? J Am Coll Cardiol. 2024;84:121‐123. 38925725 10.1016/j.jacc.2024.04.044

[54] Tardif JC , Kouz S , Waters DD , et al. Efficacy and safety of low‐dose colchicine after myocardial infarction. N Engl J Med. 2019;381:2497‐4505. 31733140 10.1056/NEJMoa1912388

[55] Nidorf SM , Fiolet ATL , Mosterd A , et al. Colchicine in patients with chronic coronary disease. N Engl J Med. 2020;383:1838‐1847. 32865380 10.1056/NEJMoa2021372

[56] Buckley LF , Libby P . Colchicine's role in cardiovascular disease management. Arterioscler Thromb Vasc Biol. 2024;44:1031‐1041. 38511324 10.1161/ATVBAHA.124.319851 PMC11047118

[57] Opstal TSJ , Hoogeveen RM , Fiolet ATL , et al. Colchicine attenuates inflammation beyond the inflammasome in chronic coronary artery disease: a LoDoCo2 proteomic substudy. Circulation. 2020;142(20):1996‐1998. doi:10.1161/CIRCULATIONAHA.120.050560 32864998

[58] Mohammadnia N , van Broekhoven A , Bax WA , et al. The effects of colchicine on lipoprotein(a) and oxidized phospholipid associated cardiovascular disease risk. Eur J Prev Cardiol. 2024;zwae355. 39478683 10.1093/eurjpc/zwae355 PMC12257107

[59] Shah B , Pillinger M , Zhong H , et al. Effects of acute colchicine administration prior to percutaneous coronary intervention: COLCHICINE‐PCI randomized trial. Circ Cardiovasc Interv. 2020;13:e008717. 32295417 10.1161/CIRCINTERVENTIONS.119.008717 PMC7169992

[60] Shah B , Smilowitz NR , Xia Y , et al. Major adverse cardiovascular events after colchicine administration before percutaneous coronary intervention: follow‐up of the colchicine‐PCI trial. Am J Cardiol. 2023;204:26‐28. 37536200 10.1016/j.amjcard.2023.07.029 PMC10947505

[61] Tong DC , Quinn S , Nasis A , et al. Colchicine in patients with acute coronary syndrome: the Australian COPS randomized clinical trial. Circulation. 2020;142(20):1890‐1900. doi:10.1161/CIRCULATIONAHA.120.050771 32862667

[62] Tong DC , Bloom JE , Quinn S , et al. Colchicine in patients with acute coronary syndrome: two‐year follow‐up of the Australian COPS randomized clinical trial. Circulation. 2021;144:1584‐1586. 34748393 10.1161/CIRCULATIONAHA.121.054610

[63] Jolly SS , d'Entremont MA , Lee SF , et al. Colchicine in acute myocardial infarction. N Engl J Med. 2024;392:633‐642. 39555823 10.1056/NEJMoa2405922

[64] Jolly SS , d'Entremont MA , Pitt B , et al. Routine spironolactone in acute myocardial infarction. N Engl J Med. 2024;392(7):643‐652. 39555814 10.1056/NEJMoa2405923

[65] Bhatt AS , Moscone A , McElrath EE , et al. Fewer hospitalizations for acute cardiovascular conditions during the COVID‐19 pandemic. J Am Coll Cardiol. 2020;76:280‐288. 32470516 10.1016/j.jacc.2020.05.038 PMC7250561

[66] Pitt B , Zannad F , Remme WJ , et al. The effect of spironolactone on morbidity and mortality in patients with severe heart failure. N Engl J Med. 1999;341:709‐717. 10471456 10.1056/NEJM199909023411001

[67] Pitt B , Remme W , Zannad F , et al. Eplerenone, a selective aldosterone blocker, in patients with left ventricular dysfunction after myocardial infarction. N Engl J Med. 2003;348:1309‐1321. 12668699 10.1056/NEJMoa030207

[68] Fiolet ATL , Poorthuis MHF , Opstal TSJ , et al. Colchicine cardiovascular trialists collaboration. Colchicine for secondary prevention of ischaemic stroke and atherosclerotic events: a meta‐analysis of randomised trials. EClinicalMedicine. 2024;76:102835. 39431112 10.1016/j.eclinm.2024.102835 PMC11490869

[69] Kelly P , Lemmens R , Weimar C , et al. Long‐term colchicine for the prevention of vascular recurrent events in non‐cardioembolic stroke (CONVINCE): a randomised controlled trial. Lancet. 2024;404:125‐133. 38857611 10.1016/S0140-6736(24)00968-1

[70] Jammoul N , Mercier G , Roubille F . Could colchicine reduce cardiovascular events in coronary artery disease without increasing all‐cause mortality: avoid optical illusions! Int J Cardiol. 2023;376:125‐126. 36716969 10.1016/j.ijcard.2023.01.064

[71] Nidorf SM , Ben‐Chetrit E , Ridker PM . Low‐dose colchicine for atherosclerosis: long‐term safety. Eur Heart J. 2024;45:1596‐1601. 38596868 10.1093/eurheartj/ehae208

[72] Opstal TSJ , Nidorf SM , Fiolet AT , et al. Drivers of mortality in patients with chronic coronary disease in the low‐dose colchicine 2 trial. Int J Cardiol. 2023;372:1‐5. 36529304 10.1016/j.ijcard.2022.12.026

[73] Ou Z , Wang F , Chen Y , et al. Comparative efficacy of colchicine and intensive low‐density lipoprotein cholesterol lowering in patients with atherosclerotic diseases receiving statins: a network meta‐analysis of randomized controlled trials. Cardiovasc Drugs Ther. 2024;1‐12. doi:10.1007/s10557-024-07622-9 39207624

[74] Ridker PM , MacFadyen JG , Thuren T , Libby P . Residual inflammatory risk associated with interleukin‐18 and interleukin‐6 after successful interleukin‐1β inhibition with canakinumab: further rationale for the development of targeted anti‐cytokine therapies for the treatment of atherothrombosis. Eur Heart J. 2020;41:2153‐2163. 31504417 10.1093/eurheartj/ehz542

[75] Broch K , Anstensrud AK , Woxholt S , et al. Randomized trial of interleukin‐6 receptor inhibition in patients with acute ST‐segment elevation myocardial infarction. J Am Coll Cardiol. 2021;77:1845‐1855. 33858620 10.1016/j.jacc.2021.02.049

[76] Ridker PM , Devalaraja M , Baeres FMM , et al. IL‐6 inhibition with ziltivekimab in patients at high atherosclerotic risk (RESCUE): a double‐blind, randomised, placebo‐controlled, phase 2 trial. Lancet. 2021;397:2060‐2069. 34015342 10.1016/S0140-6736(21)00520-1

[77] Abbate A , Trankle CR , Buckley LF , et al. Interleukin‐1 blockade inhibits the acute inflammatory response in patients with ST‐segment‐elevation myocardial infarction. J Am Heart Assoc. 2020;9(5):e014941. doi:10.1161/JAHA.119.014941 32122219 PMC7335541

[78] Abbate A , Van Tassell B , Bogin V , et al. Results of international, double‐blind, randomized, placebo‐controlled, phase IIa study of Interleukin‐1 blockade with RPH‐104 (Goflikicept) in patients with ST‐segment‐elevation myocardial infarction (STEMI). Circulation. 2024;150:580‐582. 39133774 10.1161/CIRCULATIONAHA.124.069396

[79] Myachikova VY , Maslyanskiy AL , Moiseeva OM , et al. Treatment of idiopathic recurrent pericarditis with Goflikicept: phase II/III study results. J Am Coll Cardiol. 2023;82:30‐40. 37380301 10.1016/j.jacc.2023.04.046

[80] Potere N , Bonaventura A , Abbate A . Novel therapeutics and upcoming clinical trials targeting inflammation in cardiovascular diseases. Arterioscler Thromb Vasc Biol. 2024;44:2371‐2395. 39387118 10.1161/ATVBAHA.124.319980 PMC11602387

[81] Hansson GK , Libby P , Tabas I . Inflammation and plaque vulnerability. J Intern Med. 2015;278:483‐493. 26260307 10.1111/joim.12406 PMC5082111

[82] Koenig W , Giovas P , Nicholls SJ . Combining cholesterol‐lowering strategies with imaging data: a visible benefit? Eur J Prev Cardiol. 2019;26:365‐379. 30160512 10.1177/2047487318798059

[83] West HW , Dangas K , Antoniades C . Advances in clinical imaging of vascular inflammation: a state‐of‐the‐art review. JACC Basic Transl Sci. 2023;9:710‐732. 38984055 10.1016/j.jacbts.2023.10.007 PMC11228120

[84] Antoniades C , Tousoulis D , Vavlukis M , et al. Perivascular adipose tissue as a source of therapeutic targets and clinical biomarkers: a clinical consensus statement from the European Society of Cardiology Working Group on coronary pathophysiology and microcirculation. Eur Heart J. 2023;44:3827‐3844. 37599464 10.1093/eurheartj/ehad484 PMC10568001

[85] Antonopoulos AS , Sanna F , Sabharwal N , et al. Detecting human coronary inflammation by imaging perivascular fat. Sci Transl Med. 2017;9(398):eaal2658. doi:10.1126/scitranslmed.aal2658 28701474

[86] Klüner LV , Chan K , Antoniades C . Using artificial intelligence to study atherosclerosis from computed tomography imaging: a state‐of‐the‐art review of the current literature. Atherosclerosis. 2024;398:117580. 38852022 10.1016/j.atherosclerosis.2024.117580 PMC11579307

[87] Simantiris S , Pappa A , Papastamos C , et al. Perivascular fat: a novel risk factor for coronary artery disease. Diagnostics Basel. 2024;14:1830. 39202318 10.3390/diagnostics14161830 PMC11353828

[88] Oikonomou EK , Marwan M , Desai MY , et al. Non‐invasive detection of coronary inflammation using computed tomography and prediction of residual cardiovascular risk (the CRISP CT study): a post‐hoc analysis of prospective outcome data. Lancet. 2018;392:929‐939. 30170852 10.1016/S0140-6736(18)31114-0 PMC6137540

[89] Chan K , Wahome E , Tsiachristas A , et al. Inflammatory risk and cardiovascular events in patients without obstructive coronary artery disease: the ORFAN multicentre, longitudinal cohort study. Lancet. 2024;403(10444):2606‐2618. doi:10.1016/S0140-6736(24)00596-8 38823406 PMC11664027

[90] Oikonomou EK , Antonopoulos AS , Schottlander D , et al. Standardized measurement of coronary inflammation using cardiovascular computed tomography: integration in clinical care as a prognostic medical device. Cardiovasc Res. 2021;117:2677‐2690. 34450625 10.1093/cvr/cvab286

